# Preventing Gestational Diabetes Mellitus by Improving Healthy Diet and/or Physical Activity during Pregnancy: An Umbrella Review

**DOI:** 10.3390/nu14102066

**Published:** 2022-05-14

**Authors:** Malak Kouiti, Cristian Hernández-Muñiz, Ibtissam Youlyouz-Marfak, Inmaculada Salcedo-Bellido, Juan Mozas-Moreno, José Juan Jiménez-Moleón

**Affiliations:** 1 Department of Preventive Medicine and Public Health, Universidad de Granada, 18016 Granada, Spain; m.kouiti@uhp.ac.ma (M.K.); cristianhdzm@correo.ugr.es (C.H.-M.); isalcedo@ugr.es (I.S.-B.); 2 Laboratory of Health Sciences and Technologies, Higher Institute of Health Sciences, Hassan First University of Settat, Settat 26000, Morocco; ibtissam.marfak@uhp.ac.ma; 3 Consortium for Biomedical Research in Epidemiology and Public Health (CIBERESP), 28029 Madrid, Spain; 4 Instituto de Investigación Biosanitaria (ibs.GRANADA), 18014 Granada, Spain; 5 Department of Obstetrics and Gynecology, Universidad de Granada, 18016 Granada, Spain; 6 Obstetrics and Gynecology Service, Virgen de las Nieves University Hospital, 18014 Granada, Spain

**Keywords:** gestational diabetes mellitus, dietary intervention, physical activity intervention, randomized controlled clinical trials, experimental studies, systematic reviews, meta-analysis

## Abstract

Several epidemiological studies have analyzed the effects of lifestyle modification on reducing the risk of gestational diabetes mellitus (GDM); however, their results remain inconsistent. This umbrella review aims to evaluate the effects of diet and/or physical activity interventions during pregnancy on preventing GDM. Systematic reviews and meta-analysis of randomized clinical trials reporting preventive effects of diet and/or physical activity in reducing the incidence of GDM were included from PubMed, Web of Science, Scopus and Cochrane library. Two authors independently assessed the overlapping and quality of the 35 selected reviews using AMSTAR 2. The results, although variable, tend to defend the protective role of diet and physical activity interventions separately and independently of each other in the prevention of GDM. However, the results for the combined interventions show a possible protective effect; however, it is not entirely clear because most of the analyzed meta-analyses tend to approach 1, and heterogeneity cannot be ruled out. Establishing conclusions about the most efficient type of intervention and a dose–effect relationship was not feasible given the low quality of systematic reviews (83% low to critically low) and the variability in reporting interventions. Therefore, more studies with better quality and definition of the interventions are required. The protocol was previously registered in PROSPERO as CRD42021237895.

## 1. Introduction

Gestational Diabetes Mellitus (GDM) is the most frequent metabolic disease identified during pregnancy and is a growing public health problems. GDM has been associated with both short- and long-term adverse maternal and fetal health outcomes. Newborn complications, for example, include macrosomia, hypoglycemia and birth trauma [1,2,3]. For the mother, GDM increases the risk of developing diabetes mellitus type 2 and the risk of cardiovascular diseases [4,5,6]. Risk factors associated with GDM can be divided into non-modifiable and modifiable. Age, family history of diabetes, the genetic component and race have been described as non-modifiable risk factors of GDM [7,8,9,10,11]. 

However, among the main factors associated with a high risk of GDM is weight, concretely overweight, obesity and an excessive weight gain during the pregnancy, which are factors that are potentially modifiable for GDM [9,12]. Thus, weight is very related to the diet type and the level of physical activity. In public health, factors susceptible to change improving lifestyle are very important for the prevention of diseases [13,14]. Sedentary behavior and diet with high caloric intake increase the risk of developing GDM [15,16]. In contrast, a Mediterranean diet pattern, for example, was associated with a lower risk of the disease [17].

Epidemiologic studies examining the effects of diet and physical activity on GDM prevention have increased in recent years. Several systematic reviews have been conducted regarding this subject [18,19,20]; however, their results are still inconsistent, and the most effective strategy remains unclear [21]. Some reviews defend that physical activity or diet reduces the risk of GDM [22,23]. Whereas other systematic reviews do not show a significant protective effect [24,25]. 

Several reasons may explain this lack of uniformity in the results of the systematic reviews conducted to date. The quality of the reviews, the characteristics of the evaluated diet and/or physical activity interventions, and the selection criteria used to select the studies, including the characteristics of the population selected, could help us to understand this heterogeneity. Therefore, we conducted an umbrella review to evaluate the effects of diet and physical activity interventions on the prevention of GDM, through an evidence synthesis of systematic reviews with or without meta-analysis of randomized clinical trials, evaluating the quality of the methodology of each systematic review. 

## 2. Materials and Methods

An umbrella review of systematic reviews/meta-analysis was conducted in accordance with a previous protocol registered in PROSPERO (CRD42021237895).

### 2.1. Eligibility Criteria for the Selection of Systematic Reviews

The predefined inclusion criteria for our systematic review selection were: (1) Systematic reviews/meta-analysis based on randomized controlled trials. (2) Evaluating diet and physical activity interventions, separately or in combination. (3) Including GDM as a primary or secondary outcome. (4) Published in English, Spanish, French or Arabic from the inception of the databases used for researching until December 2021. All narrative reviews, gray literature, books and book chapters and communications at conferences were excluded.

Exposure was defined as those interventions aimed at modifying lifestyle by improving diet and/or physical activity before and during pregnancy to prevent GDM compared to the usual routine care. For data synthesis, studies were grouped according to the type of intervention conducted.

### 2.2. Information Sources and Search Strategy

A literature search was conducted using the major biomedical sources, including PubMed, Scopus, Web of Science and The Cochrane Library (Cochrane Database of Systematic Reviews, Cochrane Methodology Register). In addition, the research was completed by hand-searching the references included in each selected review, and alerts were activated in PubMed and ResearchGate to stay updated. 

A primary search was performed in December 2020. The search was rerun in December 2021. No additional systematic review was included in the update as none met our inclusion criteria. 

The following terms were combined when performing the search:

- Gestational diabetes mellitus, gestational diabetes.
- Activit*, physical activity, exercise, sport, training, fitness.
- Eating behaviors, feeding behaviors, eating habits, food habits, dietary habits, feeding patterns, dietary pattern, diet.
- Systematic review, meta-analysis.
- Diabetes mellitus type 1, diabetes mellitus type 2, T2D, DM2, treatment.

For example, in PubMed, a broad search was used combining natural language terms and MeSH terms, the following search equation was applied: ((“gestational diabetes mellitus” OR “gestational diabetes”) AND ((“physical activity” OR Activit* OR exercise OR sport OR training OR fitness) OR (“eating behaviors” OR “feeding behaviors” OR “eating habits” OR “food habits” OR “dietary habits” OR “feeding patterns” OR “dietary pattern” OR diet))) AND (“systematic review” OR meta-analysis) NOT (“diabetes mellitus type 1” [Mesh] OR “diabetes mellitus type 2” [Mesh]). The same keywords were used in all databases, adapting the equation to the form required in each. Further details about search strategy are provided in Supplementary Material, (Tables S1–S3).

### 2.3. Study Selection and Extraction Data

Two members of the research team (MK and CHM) performed the search and selection of systematic reviews and meta-analyses independently. By evaluating the title and the abstract, a first screening of the reviews that met the selection criteria was made. If there were any doubts or disagreements between the two researchers, the full text was read. Persistent disagreements were resolved through the advice of a third investigator (JJJM). Data extraction was conducted individually and independently by the same two researchers who conducted the first search and selection of systematic reviews with or without meta-analysis (MK and CHM). Information was stored in a structured way using a database. 

The relevant information included was the following: author, publication year, journal and its impact factor according to Journal Citation Reports; number of studies included in its systematic reviews; databases used in the search; publication years included in the review; selection criteria of the systematic review; global sample size, characteristics of the interventions related to diet and physical activity as frequency, intensity and length of sessions; tools for evaluating the quality of the studies included in the reviews (Cochrane Handbook, Jadad scale and GRADE); analysis of heterogeneity of the studies included in the reviews, risk of publication bias assessment and review’s funding sources. 

In the same way, the association measures used to evaluate the magnitude of the association were extracted and analyzed: total relative risk (RR), odds ratio (OR) estimated or risk difference (RD) and their 95% confidence intervals.

### 2.4. Quality Assessment

AMSTAR-2 was used to evaluate the quality of the systematic reviews included in the umbrella. AMSTAR is a specific tool developed by B.J. Shea et al. to assess the quality of systematic reviews of randomized controlled clinical trials for the evaluation of healthcare interventions [26,27]. AMSTAR-2 improves the characteristics of AMSTAR, allowing a deep evaluation of systematic reviews and both randomized and non-randomized studies. 

The first items are dedicated to assessing the research question according to the PICO structure, the selection criteria, the existence of a previously registered protocol, and the justification of the type of design of the included studies in the systematic review. The remaining items attempt to assess methodological aspects related to the interpretation of the results and their discussion, in addition to the evaluation of the risks of bias and the analysis of heterogeneity [28]. Of the 16 items, seven are considered critical weaknesses: items 2, 4, 7, 9, 11, 13 and 15. The following describes how the evaluation of such items was conducted: 

(1) Item 2: The systematic review must explicitly report the use of a previous protocol established before its implementation. If the protocol exists but has not been registered, the answer to this item is a “partial yes”. 

(2) Item 4: Evaluates the study search conducted. An adequate bibliographic search must include at least the following criteria: use of at least two databases, reporting the search strategy, keywords and restrictions that have been applied in the databases. When these criteria are met, a “partial yes” evaluation is obtained. The “yes” rating requires searching in the references of the selected articles, the gray literature, consulting experts and conducting the search within 24 months after the protocol and no more than 6 months prior to the acceptance of work. 

(3) Item 7: The mention of excluded studies allows obtaining a “partial yes” and a “yes” requires explaining why they are excluded. 

(4) Item 9: Assess the risk of bias of the selected studies (RCT) using adequate tools. Blinding and randomization masking are required at least for a ‘partial yes’ (Cochrane manual, GRADE or Jadad scale, for example). A “yes” qualification requires that the authors evaluate the generation of a random sequence to allocate the participants to the comparison groups. 

(5) Item 11: Qualified as “yes” when the meta-analysis is justified, a random-effects model is used in the combination of data and adjusted for heterogeneity if necessary. Furthermore, the causes of heterogeneity are investigated. 

(6) Item 13: Considering the risk of bias in the interpretation of results, including only studies with a low risk of bias or discussing the possible impact on the results, allows a “yes” classification. 

(7) Item 15: It is evaluated as “yes” when the publication bias is explicitly reported using a funnel graph or the performance of the Egger test. AMSTAR-2 tool was applied by two researchers independently (MK and CHM). The doubts and disagreements that arose were discussed and resolved by a third investigator (JJJM).

After assessing the quality of the systematic reviews included in our umbrella, the results were stratified according to the following cut-off points for AMSTAR-2: (1) Critically low quality: the systematic review does not meet more than one critical item, regardless of the existence or not of non-critical weaknesses. (2) Low quality: the systematic reviews does not meet a critical weakness, meeting or not the rest of the items identified as non-critical weakness. (3) Moderate quality: the systematic review complies with all critical elements and does not meet more than one non-critical weakness. (4) High quality: when all critical elements are met, and there is only one non-critical weakness at most [28].

### 2.5. Overlapping Synthesis

When two or more systematic reviews investigated the same type of exposure and the risk of GDM, the primary studies included in each review should overlap for the coinciding time periods. In the present umbrella, the evaluation of the overlap was conducted according to the method described by Pieper and Okoth [29,30]. Examination of overlap was done for each intervention (physical activity, diet and mixed approach with both interventions). 

In addition, reviews were distributed by year of publication (reviews published before 2015 and since 2015). To assess overlapping, the characteristics of the population were also considered (pregnant women in general and pregnant women at high risk of suffering GDM). For reviews that have an update, only the latest version was included in the overlap assessment [25,31]. In systematic reviews where overlapping was assessed, a ‘Citation Matrix’ was performed (Cross Tabulation Chart), including systematic reviews in columns, and primary studies in rows were performed [32]. 

This matrix of citations made it possible to measure the overlap value with a method called “Corrected Covered Area” (CCA) [30]. This procedure allows quantifying the percentage in degrees of overlap between two or more reviews, helping in the decision-making process on how to handle the overlap when it is present [30].

The equation to calculate the CCA is: (N-r)/(rc-r); Where “N” (grand total) is the value that includes the number of primary studies evaluated in each of the systematic reviews included, that is, the number of boxes selected in the citation matrix; “R” (rows) is the number of rows of the primary studies investigated in the systematic reviews; “C” (columns) is the number of columns corresponding to the systematic reviews included in the overlap assessment. The CCA expressed as a percentage allows a classification of the degree of the overlap as “very high” when the CCA is greater than 15%; “High” if the CCA has a value between 11% and 15%; “Medium” when the CCA obtains the value of 6–10%; and finally, “low” when the value is 0–5% [30].

### 2.6. Data Synthesis

Data from systematic reviews and meta-analyses that met the selection criteria were analyzed. A synthesis of the different interventions evaluated in the RCTs included in the systematic reviews was conducted. 

## 3. Results

### 3.1. Literature Search

From the four bibliographic sources selected for searching, 693 articles were retrieved (PubMed *n* = 222; Web of Science *n* = 209; Scopus *n* = 150; and Cochrane library *n* = 112). A total of 189 articles were eliminated due to being duplicates, and 448 were definitively excluded after the title and abstract screening. Accordingly, the full text of 56 papers was evaluated. A final 34 systematic reviews met the selection criteria and were included in our umbrella. The reasons for the exclusion of the papers not selected by full-text assessment can be consulted in Supplementary Material, (Supplementary Table S4). 

**Figure 1:**
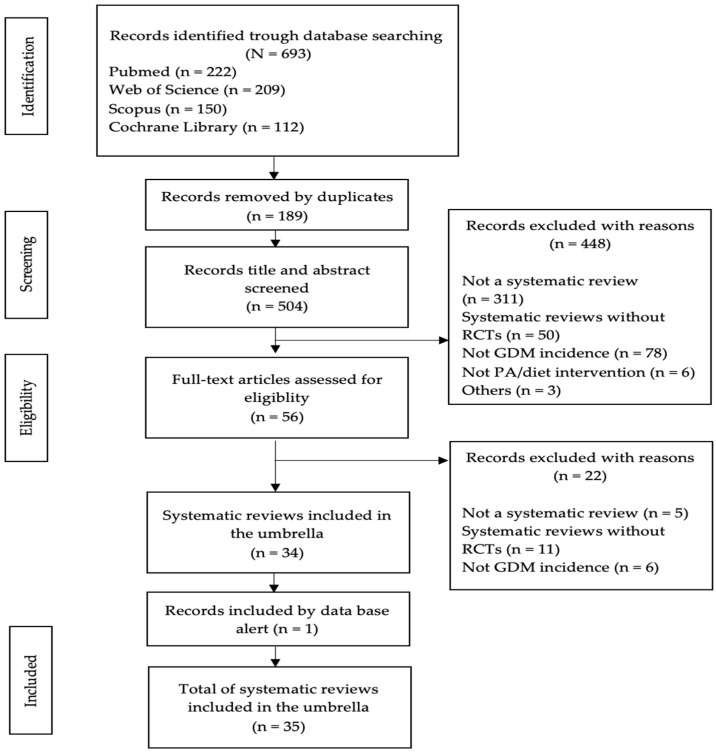
Flow chart: Systematic review selection process. RCTs: Randomized controlled trials; GDM: Gestational Diabetes Mellitus; and PA: Physical Activity.

One review was identified thanks to the alerts activated in PubMed and the research social network ResearchGate. Thus, 35 systematic reviews were finally included in this umbrella review. All, except for three systematic reviews [33,34,35], also include a meta-analysis of the data of the individual clinical trials in each systematic review. Figure 1 summarizes the process applied for the selection of systematic reviews and meta-analyses included in this umbrella.

According to the intervention evaluated in each systematic reviews, the whole of the systematic reviews were classified into three groups: (a) systematic reviews about physical activity only: *n* = 16 (45.7%) [19,24,33,36,37,38,39,40,41,42,43,44,45,46,47,48]; (b) systematic reviews containing information about diet exclusively: *n* = 4 (11.4%) [25,34,49,50]; and (c) systematic reviews with information about both types of interventions, diet and physical activity: *n* = 15 (42.9%) [18,20,22,23,31,35,51,52,53,54,55,56,57,58,59]. 

### 3.2. Quality Assessment of the Systematic Reviews 

Of the 35 systematic reviews selected for this umbrella, 19 (54.2%) were classified as critically low quality and 10 (28.6%) as low quality. The number of systematic reviews of medium and high quality was three for each category. More information about the quality evaluation can be consulted in, Supplementary Figure S1.

Three items were evaluated only in 32 systematic reviews that included a meta-analysis. Item 11: Appropriate meta-analysis methods; A total of 27 (84.4%) meta-analyses used an appropriate meta-analysis method for the statistic combination. Item 12: Assessing the potential impact of bias risk on results; 31 (93.3%) meta-analyses assessed the potential impact of bias risk on results. Item 15: Assessment of the presence and probable impact of publication bias; 23 (71.9%) meta-analyses investigated the presence of publication bias and assessed its possible impact. Only 25.7% of systematic reviews registered a protocol before conducting the systematic review (Item 2 from AMSTAR-2 tool), and 54.3% did not provide a list of the original studies excluded from the reviews as well as its justification (Item 7). 

**Figure 2:**
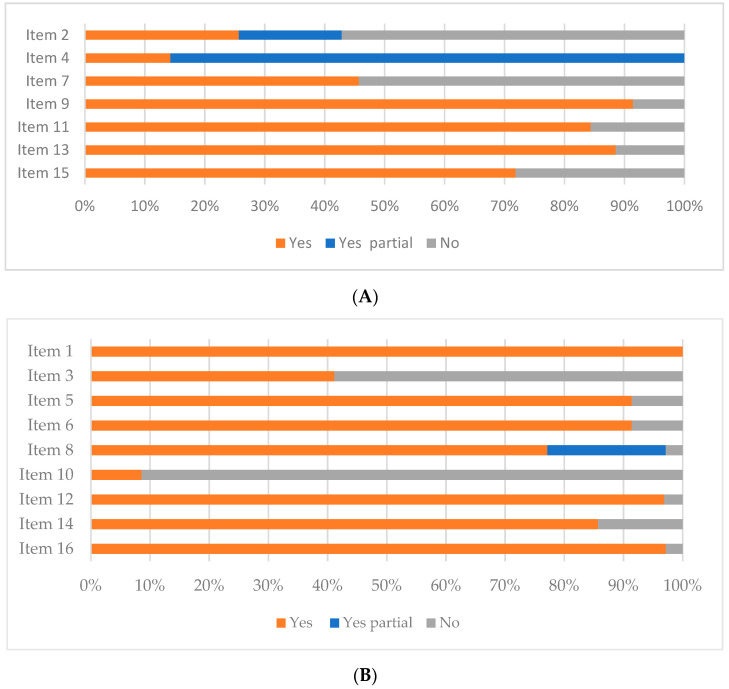
Accomplishment of systematic review with AMSTAR-2 items. (**A**) Critical items. (**B**) Non-critical items. Critical items: Item 2: Previous protocol review; Item 4: Adequate literature search; Item 7: Excluded studies justification; Item 9: Bias risk of individual studies included; Item 11: Appropriate meta-analysis methods; Item 13: Consideration of the bias risk in the interpretation of the review results; Item 15: Assessment of the presence and probable impact of publication bias. Non-critical items: Item 1: Research questions and inclusion criteria include PICO components; Item 3: Explaining decision about the study designs to include in the review; Item 5: Study selection performed in duplicate; Item 6: Data extraction performed in duplicate; Item 8: Describing included studies with sufficient detail; Item 10: Reporting the sources of funding for the studies included in the review; Item 12: Assessing the potential impact of bias risk on results; Item 14: Satisfactory explanation and discussing any observed heterogeneity in the review results; and Item 16: Potential sources of conflict including any funding received.

The presence and probable impact of the publication bias was not assessed in 28.1% of the systematic reviews included in this umbrella (Item 15). Figure 2A shows the results for the seven critical items from AMSTAR-2 tool. Regarding not critical items, the quality of the included systematic review was good except for the decision about study designs and the consideration of the sources of funding of the studies included in the reviews—items 3 and 10, respectively (Figure 2B).

### 3.3. Overlapping between Reviews

Analysis of the overlapping was performed by groups of systematic reviews determined by: (a) The year of publication, differencing between systematic reviews published before 2015 and from 2015. (b) The type of the evaluated interventions: “physical activity”, “diet” or “mixed interventions”. (c) Characteristics of the study population according to risk pregnancy: “pregnant women at high risk” and “pregnant women in general”.

The overlapping between systematic reviews was classified as very high for all the comparisons performed, with a CCA of 19.3% to 37.5% (See Table 1). Despite this, all 35 reviews have been maintained. This decision is explained by the high heterogeneity in the original studies included in each review. For example, for the 11 systematic reviews on physical activity interventions in general pregnant women published since 2015, 35 original RCTs studies were included. However, the 35 RCTs are not used in all 11 reviews; 10/35 were included only once, and 13/35 were used in half of the reviews. It should be noted that sometimes different articles from one study can be used. More details about the overlapping assessment are provided in Supplementary Material, (Tables S5–S9). 

### 3.4. Main Results

#### 3.4.1. Interventions

The beginning and the end of interventions differed between the RCTs involved in systematic reviews included in our umbrella. Nevertheless, lifestyle interventions mostly started before 20 week’s gestation and lasted until 34–37 weeks of gestation or until delivery.

Generally, physical activity interventions consisted of educational recommendations on physical activity plus a group session or, less often, an in-home session. These interventions mainly include an aerobic activity, muscle strength exercise, resistance and balanced exercises. The warm-up and cool-down parts of a session usually consisted of walking and stretching activities. Cycling, swimming and pelvic floor exercise was recommended often as well. In most of interventions, the intensity of exercise was light to moderate or moderate. The frequency of sessions was around three times per week and sometimes reached five times per week. The duration of each session can vary from 35 min to 60 min [19,24,33,36,37,38,39,40,41,42,43,44,45,46,47,48].

Regarding diet, the interventions consisted of recommendations and advice made through face-on-face sessions and less frequently through group sessions, completed by a phone call and/or written support. The frequency of visits was very different between trials, with a fluctuation of three to ten visits per participant. Usually, the interventions were realized by a dietitian and sometimes by a food technologist. Regarding interventions related to diet, healthy eating was promoted, especially through reducing energy intake, restricting a high glucose intake, dietary conduct and encouraging a high fiber intake [22,23,25,51,52]. 

Except for Zhang et al., the recommendation was a Mediterranean diet with a sizeable intake of virgin olive oil, fruits and vegetables, nuts, moderate to high fish consumption and a low intake of meat [49]. Respecting combined interventions, advice provided during the visits was oriented to physical activity and diet simultaneously. Counseling was conducted mainly by a nutritionist or dietitian and sometimes accompanied or conducted by a food technologist, physiotherapists or nursing staff. 

The details regarding the frequency of visits and physical activity sessions do not change from those mentioned above. The recommendations continue along the same lines: encouraging moderate physical activity at least three to five times/week and favoring a healthy and balanced diet with a low glucose intake and restricted energy consumption. In some trials, the interventions were completed by individual follow-up and personalized monitoring [23,31,59]. 

Different guidelines were used for elaborating interventions and advice. Those mentioned include the Institute of Medicine (IOM) guidelines, the American College of Obstetricians and Gynecologists guidelines for gestational weight gain, Health Canada guidelines, Prenatal nutrition guidelines, official National Dietary recommendation and Danish recommendations [22,23,31,35,55]. More details are provided in the Supplementary Material (Tables S10–S12).

#### 3.4.2. Prevention of GDM

All the meta-analyses expressed the magnitude of the association between physical activity interventions, dietary interventions or mixed interventions and the risk of GDM as relative risk (RR) or odds ratio (OR), except for the meta-analysis of Oostdam et al. [59] that used a risk difference. To describe the results, we respected the measures used by each one of the systematic reviews.

(a) Physical activity intervention

**Figure 3:**
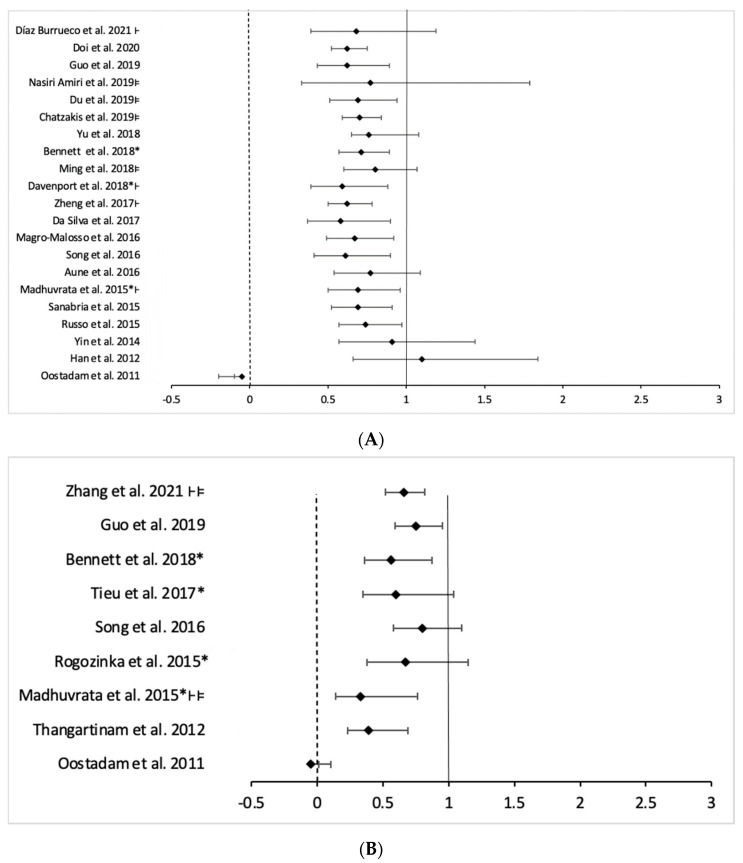

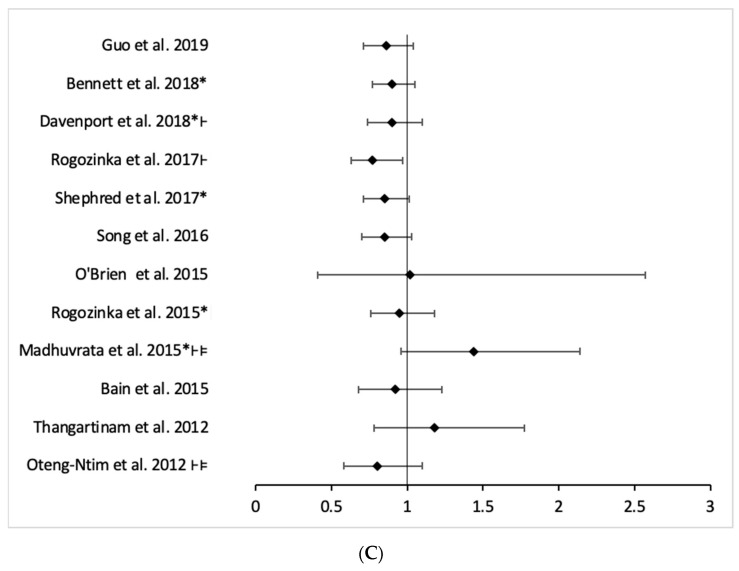
Forest plot of association of lifestyle intervention in reducing the risk of GDM. (**A**) Physical activity intervention. (**B**) Diet intervention. (**C**) Mixed intervention. * Moderate to high-quality review; ⊦: Odds Ratio; and ⊧: Women at high risk.

Most reviews show a possible preventive effect of physical activity interventions in reducing the risk of GDM, although it does not always show a statistically significant effect [23,37,59]. Although systematic reviews with moderate to high quality, such as Davenport et al. (2018) and Bennett et al. (2018), highlight that it reduces the incidence of GDM [18,39] (Table 2 and Figure 3A).

(b) Diet intervention

The protective effects of only diet intervention were very variable between reviews. The Mediterranean diet can have a significant effect (OR 0.66, 95% CI 0.52–0.82, I^2^ = 0) [49].

Diet as the lone intervention designed to reduce gestational weight gain also has a significant effect on the prevention of GDM but with a high degree of heterogeneity (RR 0.56, 95% CI 0.36–0.87, I^2^ = 53%; *p* = 0.3) [22] (Table 3 and Figure 3B). Other studies suggest that dietary consulting maybe reduce the risk of GDM in comparison with usual care, and no clear difference between low and moderate to high glycemic intake was observed [25,59]. One systematic review notes that diet had a significant protective effect only in obese and overweight pregnant women [55].

(c) Mixed intervention

When the participant received both interventions; diet and physical activity, four moderate to high-quality reviews showed a possible protective effect in reducing GDM risk (no statistically significant difference) although this effect is less clear (RR 0.90, 95% CI 0.77–1.05; I^2^ = 33%; *p* = 0.072) [22], (OR 0.90, 95% CI 95% 0.74–1.10; I^2^ = 30%; *p* = 0.09) [18], (RR 0.85, 95% CI 0.71–1.01; I^2^ = 42%; *p* = 0.03) [31], (RR 0.95, 95% CI 0.76–1.18; I^2^ = 23%; *p* = 0.21) [55]. Regardless of their quality, the CI for ORs and RRs include the value 1 in the most of the meta-analyses showing a moderate heterogeneity (Table 4 and Figure 3C).

### 3.5. Results from Studies with GDM as Not a Principal Outcome

We analyzed six reviews that show a possible protective effect in reducing the risk of GDM in each one of the interventions (Table 5). The results for these systematic reviews are very similar to those using GDM as the primary outcome. Regarding physical activity, a possible significant effect was observed (RR 0.61, 95% CI 0.41–0.90) [41]. Likewise, diet intervention based on a balanced nutritional regimen with a restriction of 2000 kcal/day (RR 0.39, 95% CI 0.23–0.69; I^2^ = 21%; *p* = 0.001) [58]. When participants can receive both interventions, the effect of the intervention is less clear (RR 1.18, 95% CI 0.78–1.77; I2 = 0%; *p* = 0.04) [58] and (OR 0.80, 95% CI 0.58–1.10; I^2^ = 62%; *p* = 0.002) [57].

## 4. Discussion

The present umbrella review proposes a synthesis of available scientific evidence on lifestyle modification through interventions based on diet and/or physical activity in the prevention of GDM using systematic reviews and meta-analyses of randomized controlled clinical trials.

Most reviews, regardless of their quality, tend to support that physical activity interventions can reduce the risk of GDM. The effectiveness of physical activity may be restricted by the accomplishment of some criteria: (a) delivering interventions in a healthcare facility [36]; (b) interventions took place early in pregnancy [33]; (c) achieving at least 600 MET-min/week of moderate-intensity [18]; or (d) only water exercise [48]. The incidence of GDM may decrease with diet intervention. However, establishing conclusions about the most effective dietary pattern can be difficult because of the differences in the dietary advice provided [34]. 

Contrary to expectations, the effect of the combined intervention is unclear or less effective than physical activity or diet alone. This may be because most meta-analyses analyzed tend to approach 1 (without showing significant statistical difference). This difference likely results from variability in the conception of interventions, their duration and other factors perhaps associated with the design of the studies and diet and physical activity patterns assessment. The quality of the RCTs included may also affect the results obtained, and this limitation cannot be discarded [48].

Variability in the descriptions of the interventions made it difficult to draw firm conclusions according to the most efficient type of activity. Similarly, in this research work, making clear recommendations and providing dose–effect analysis was not feasible for many reviews [34,35,51]. That is why it is important to establish, propose and investigate the types of interventions that are more efficient in order to have clear results.

To compare our results, only one overview was found. In contrast to our outcomes, their results show an unknown benefit for physical activity alone and diet alone interventions, although they also suggested a possible beneficial effect of combined diet and exercise [21]. It should be noted that their review was performed only for nine Cochrane reviews.

Regarding the strengths of this research: (1) To our knowledge, this umbrella is one of the few works that provide an exhaustive analysis of the three types of interventions “physical activity”, “diet” or “mixed intervention” in the prevention of GDM. (2) It was developed according to the protocol previously registered in PROSPERO. (3) An exhaustive investigation was conducted in the four most important electronic databases without date restrictions. (4) To reduce the probable bias at the time of the search, the selection of the articles, the data extraction (such as the quality assessment and the measurement of the overlap) were conducted independently by two investigators.

Regarding the limitations of the umbrella: (1) The variability, poor quality and lack of sufficient details describing intervention with respect to the type of diet, type of exercise, duration of the intervention and the intensity with which each intervention was conducted. This made it difficult to expose certain information regarding elements in the general review. This is a frequent weakness in systematic reviews that have been conducted in such a way that they focus on dietary intervention or are based on the promotion of physical activity without delving into the exact type of intervention. The effects of an intervention focused on caloric restriction do not have to be the same as those of an intervention aimed at promoting the Mediterranean diet. 

(2) In relation to the above limitation, and as a limitation of the systematic reviews, we note the relatively low quality of systematic reviews conducted to date. For this reason, the interpretation of obtained results was conducted considering their quality.

Using the current findings, it is difficult to establish a well-defined protocol or provide practical recommendations to prevent GDM based on a comprehensive description of the type of physical activity and its intensity, as well as the type of diet and its main characteristics. Even so, it is clear that scientific evidence and WHO recommendations support the benefits of healthy lifestyles, improving physical activity and eating a balanced diet in the prevention of diseases, including GDM. However, this umbrella review of systematic reviews and meta-analyses provides scientific material that summarizes the current available data to facilitate its accessibility by practitioners and other scientists.

## 5. Conclusions

The previously available systematic reviews analyzing the relationship between physical activity and/or diet were of low quality. Moreover, the definitions of interventions were heterogeneous.

The results of the systematic reviews, although variable, tend to defend the protective role of diet, such as a Mediterranean diet and physical activity, such as three to five sessions a week of 30 min duration and moderate intensity, in preventing GDM. However, the protective effects of a mixed intervention with both are not completely clear. Furthermore, there is insufficient evidence of high quality to determine that combined interventions have a protective effect.

Establishing conclusions on the most efficient type of intervention and a dose–effect relationship has not been feasible given the high variability in the description of the interventions and the low quality of the revisions. Our results highlight the need to perform more clinical trials of better quality and approach interventions and systematic reviews with quality corresponding to the current standards.

## Figures and Tables

**Table 1:** Overlapping between reviews.

Overlapping	N	CCA	Classification
**Physical activity as only intervention**			
Reviews of RCTs with pregnant women in general published since 2015	11	26.28%	Very high
Reviews of RCTs with high-risk women published since 2015	6	19.26%	Very high
Reviews of RCTs with pregnant women in general published before 2015	3	37.5%	Very high
**Diet as only intervention**	4	25.39%	Very high
**Mixed intervention**	5	36.49%	Very high

(N = Number of reviews included in the overlapping assessment; CCA = Corrected Covered Area).

**Table 2:** Systematic reviews finding including physical activity intervention in reducing GDM.

Systematic Review ID	RCTs Number	Participant Included in Intervention and Control Group (*N*/*n*)	Association Measurement	I^2^ (*p*)	Quality (Amstar 2)	
Oostdam et al., 2011 [59]	3	125/113	RD −0.05 (−0.20–0.10)	66 (0.05)	Critically low	
Han et al., 2012 [47]	5	437/389	RR 1.10 (0.66–1.84)	0 (0.37)	Low	
Yin et al., 2014 [46]	5	497/450	RR 0.91 (0.57–1.44)	26 (0.25)	Critically low	
Russo et al., 2015 [45]	10	569/520	RR 0.74 (0.57–0.97)	12 (0.33)	Critically low	
Sanabria-Martínez et al., 2015 [44]	8	N.A.	RR 0.69 (0.52–0.91)	0 (0.61)	Critically low	
Madhuvrata et al., 2015 * [23]	3	76/76	OR 0.77 (0.33–1.79)	0 (0.53)	Moderate	
Aune et al., 2016 [19]	12	9804 **	RR 0.69 (0.50–0.96)	30.2 (0.15)	Critically low	
Song et al., 2016 [20]	10	4161 **	RR 0.77 (0.54–1.09)	N.A.	Critically low	
Da Silva et al., 2017 [43]	10	1883/1907	RR 0.67 (0.49–0.92)	33 (0.14)	Critically low	
Zheng et al., 2017 [24]	7	550/563	OR 0.62 (0.43–0.89)	37 (0.19)	Critically low	
Ming et al., 2018 * [42]	9	1472/1509	RR 0.58 (0.37–0.90)	46 (0.07)	Low	
Davenport et al., 2018 [18]	27	7568/7198	OR 0.62 (0.52–0.75)	0 (0.51)	High	
Bennett et al., 2018 [22]	10	2981 **	RR 0.62 (0.50–0.78)	0 (0.90)	Moderate	
Yu et al., 2018 [39]	6	651/719	RR 0.59 (0.39–0.88)	46 (0.11)	Critically low	
Chatzakis et al., 2019 * [40]	14	575/589	RR 0.80 (0.60–1.07)	30	Low	
Du et la., 2019 * [38]	13	550/572	RR 0.71 (0.57–0.89)	0 (0.52)	Low	
Makaruk et al., 2019 [33]	10	1747/2013	N.A.	N.A.	Critically low	
Nasiri-Amiri et al., 2019 * [37]	8	727/714	RR 0.76 (0.65–1.08)	50 (0.05)	Critically low	
Guo et al., 2019 [51]	19	5883 **	RR 0.70 (0.95–0.84)	N.A.	Critically low	
Doi et al., 2020 * [36]	11	722/745	RR 0.69 (0.51–0.94)	23.2 (0.02)	Low	

* Population = women at high risk; ** Total sample size; N.A. = Not available; OR = odds ratio; RR = relative risk; RD = risk difference; *N* = exposed sample size; *n* = no exposed sample size; and I^2^(*p*) = heterogeneity test (*p*-value).

**Table 3:** Systematic reviews finding including diet intervention in reducing GDM.

Systematic Review ID	RCTs Number	Participant Included in Intervention and Control Group (*N*/*n*)	Association Measurement	I^2^ (*p*)	Quality (Amstar 2)
Oostdam et al., 2011 [59]	7	449/364	RD −0.05 (−0.10–−0.01)	41 (0.12)	Critically low
Madhuvrata et al., 2015 * [23]	3	202/207	OR 0.33 (0.14–0.76)	26 (0.26)	Moderate
Rogozińska et al., 2015 [55]	6	725/754	RR 0.67 (0.38–1.15)	52 (0.06)	Moderate
Song et al., 2016 [20]	5	1279 **	RR 0.80 (0.58–1.10)	-	Critically low
Tieu et al., 2017 [25]	11	628/652	RR 0.60 (0.35–1.04)	56 (0.07)	High
Bennett et al., 2018 [22]	9	3388 **	RR 0.56 (0.36–0.87)	53 (0.03)	Moderate
Lamminpää et al., 2018 [34]	15	N.A.	N.A.	N.A.	Critically low
Guo et al., 2019 [51]	11	2838 **	RR 0.75 (0.59–0.95)	N.A.	Critically low
Zhang et al., 2020 *** [49]	2	911/937	OR 0.66 (0.52–0.82)	0 (0.85)	Critically low

* Population = women at high risk; ** Total sample size; *** Mediterranean diet; N.A. = Not available; OR = odds ratio; RR = relative risk; *N* = exposed simple size; *n* = no exposed simple size; and I^2^(*p*) = heterogeneity test (*p*-value).

**Table 4:** Systematic reviews finding including mixed intervention in reducing GDM.

Study ID	RCTs Number	Participant Included in Intervention and Control Group (*N/n*)	Association Measurement	I^2^(*p*)	Quality (Amstar 2)
Bain et al., 2015 [56]	13	1903/1841	RR 0.92 (0.68–1.23)	43.13 (0.06)	Low
Madhuvrata et al., 2015 * [23]	6	562/526	OR 1.44 (0.96–2.14)	0 (0.93)	Moderate
Rogozińska et al., 2015 [55]	12	2399/2346	RR 0.95 (0.76–1.18)	23 (0.21)	Moderate
Song et al., 2016 [20]	14	6047 **	RR 0.85 (0.70–1.03)	N.A.	Critically low
Shepherd et al., 2017 [31]	19	3353/3280	RR 0.85 (0.71–1.01)	42 (0.03)	High
Davenport et al., 2018 [18]	22	575/550	OR 0.90 (0.74–1.10)	30 (0.09)	High
Bennett et al., 2018 [22]	22	7274 **	RR 0.90 (0.77–1.05)	33 (0.072)	Moderate
Guo et al., 2019 [51]	18	7024 **	RR 0.86 (0.71–1.04)	N.A.	Critically low

* Population = women at high risk; ** Total sample size; N.A. = Not available; OR = odds ratio; RR = relative risk; N = exposed simple size; n = no exposed simple size; and I^2^(*p*) = heterogeneity test (*p*-value).

**Table 5:** Systematic reviews finding including GDM as a secondary outcome.

Systematic Reviews with GDM as Not the Principal Outcome					
	RCTs Number	Participant Included in Intervention and Control Group (*N/n*)	Association Measurement	I^2^ (*p*)	Quality (Amstar 2)
**Physical activity**					
Magro-Malosso et al., 2017 * [41]	7	623/727	RR 0.61 (0.41–0.90)	-	Critically low
Díaz-Burrueco et al., 2021 [48]	5	782/1091	OR 0.68 (0.39–1.19)	-	Low
**Diet**					
Thangaratinam et al., 2012 [58]	3	409 **	RR 0.39 (0.23–0.69)	21 (0.001)	Critically Low
**Mixed intervention**					
Rogozinska et al., 2017 [53]	31	5710/5408	OR 0.77 (0.63–0.94)	38 (0.02)	Low
O’brien et al., 2016 [54]	2	243 **	RR 1.02 (0.41–2.57)	-	Critically Low
Thangaratinam et al., 2012 [58]	6	1233 **	RR 1.18 (0.78–1.77)	0 (0.44)	Critically Low
Oteng-Ntim Et al., 2012 * [57]	6	526/491	OR 0.80 (0.58–1.10)	62 (0.002)	Critically Low

(* Pregnant women at high risk; and ** Total sample size).

## Data Availability

All data generated or analyzed during this study are included in the manuscript and its Supporting Information files.

## Supplementary Materials

The following are available online at https://www.mdpi.com/article/10.3390/nu14102066/s1, Tables S1–S3: Search strategy, Table S4: Excluded studies and justification, Figure S1: Quality of included reviews assessed by Amstar 2, Tables S5–S9: Overlapping assessment, Table S10: Description of physical activity intervention in included systematic reviews, Table S11: Description of diet intervention in included systematic reviews, Table S12: Description of mixed intervention in included systematic review.
