# Supplementary material for: Preventing Gestational Diabetes Mellitus by Improving Healthy Diet and/or Physical Activity during Pregnancy: An Umbrella Review
Source: Nutrients. 2022 May 14;14(10):2066. doi: 10.3390/nu14102066 (PMC9144260; doi:10.3390/nu14102066)
Supplement: Supplementary file 1 [file nutrients-14-02066-s001.zip › nutrients-1716821-supplementary.pdf]

Table S1. Search strategy: Pubmed

Search	Query	Results
#1	"gestational diabetes mellitus" OR "gestational diabetes"	**18 197**
#2	"physical activity" OR Activit* OR exercise OR sport OR training OR fitness	**5 507 610**
#3	"eating behaviors" OR "feeding behaviors" OR "eating habits" OR "food habits" OR "dietary habits" OR "feeding patterns" OR "dietary pattern" OR diet	**562 756**
#4	"systematic review" OR metaanalysis	**308 075**
#5	"diabetes mellitus, type 1"[Mesh]OR "diabetes mellitus, type 2"[Mesh]	**201 418**
#6	#2 OR #3	**5 906 983**
#7	#1 AND #6 AND #4	**254**
#8	#7 NOT #5	**222**

Table S2. Search strategy: Web of Science

Search	Query	Results
#1	TS=("gestational diabetes mellitus" OR "gestational diabetes")	**20 078**
#2	TS=("physical activity" OR Activit* OR exercise OR sport OR training OR fitness)	**6 194 749**
#3	TS=("eating behaviors" OR "feeding behaviors" OR "eating habits" OR "food habits" OR "dietary habits" OR "feeding patterns" OR "dietary pattern" OR diet)	**555 519**
#4	TS=("systematic review" OR metaanalysis)	**376 280**
#5	TS=("diabetes mellitus type 1" OR "diabetes mellitus type 2" OR T2D OR DM2 OR treatment)	**5 330 191**
#6	#2 OR #3	**6 619 797**
#7	#1 AND #6 AND #4	**252**
#8	#7 NOT #5	**209**

Table S3. Search strategy: Cochrane library

Search	Query	Results
#1	"gestational diabetes mellitus" OR "gestational diabetes"	**2 656**
#2	"physical activity" OR Activit* OR exercise OR sport OR training OR fitness	**302 579**
#3	"eating behaviors" OR "feeding behaviors" OR "eating habits" OR "food habits" OR "dietary habits" OR "feeding patterns" OR "dietary pattern" OR diet	**66 385**
#4	"systematic review" OR metaanalysis	**24 332**
#5	#**1** AND (#**2** OR #**3**) AND #**4**	**131**

Table S4. Excluded studies and justification

Reason for exclusion	Authors	Title
Not suitable study design (Does not include RCTs)	Egan et al. 2020	A core outcome set for studies of gestational diabetes mellitus prevention and treatment
	Hasbullah et al. 2020	Factors associated with dietary glycemic index and glycemic load in pregnant women and risk for gestational diabetes mellitus
	Morales-Suarez-Varela et al. 2020	Maternal Physical Activity During Pregnancy and the Effect on the mother and Newborn: A Systematic Review
	Raghavan et al. 2019	Dietary patterns before and during pregnancy and maternal outcomes: a systematic review
	Mijatovic-Vukas et al. 2018	Associations of Diet and Physical Activity with Risk for Gestational Diabetes Mellitus: A Systematic Review and Meta-Analysis
	Carolan-Olah et al. 2017	A systematic review of interventions for Hispanic women with or at risk of Gestational diabetes mellitus (GDM)
	Donazar-Ezcurra et al. 2017	Primary prevention of gestational diabetes mellitus through nutritional factors: a systematic review
	Mijatovic 2017	Dietary Intake and Physical Activity Predictors of Gestational Diabetes: A Systematic Review
	Tieu et al. 2017	Interconception care for women with a history of gestational diabetes for improving maternal and infant outcomes
	Donazar-Ezcurra et al. 2017	Primary prevention of gestational diabetes mellitus through nutritional factors: a systematic review
	Tobias et al. 2011	Physical activity before and during pregnancy and risk of gestational diabetes mellitus: A meta-analysis
Not a systematic review	Halperin et al. 2021	The role of lifestyle interventions in the prevention of gestational diabetes
	Juan et al. 2020	Prevalence, Prevention, and Lifestyle Intervention of Gestational Diabetes Mellitus in China
	Šćepanović et al. 2020	The effect of maternal exercise on maternal and foetal health in obese pregnant women [Učinek telesne dejavnosti pri nosečnicah z debelostjo na zdravje nosečnice in novorojenčka]
	Mottola et al. 2016	Role of Exercise in Reducing Gestational Diabetes Mellitus
	Zhang et al. 2016	Risk factors for gestational diabetes: is prevention possible?

Not GDM outcomes or women diagnosed with GDM	Li et al. 2021	Effects of lifestyle intervention on long-term risk of diabetes in women with prior gestational diabetes: A systematic review and meta-analysis of randomized controlled trials
	Yaping et al. 2020	A meta-analysis of the effects of resistance training on blood sugar and pregnancy outcomes
	Xu et al. 2020	Influence of low-glycemic index diet for gestational diabetes: a meta-analysis of randomized controlled trials
	Laredo-Aguilera et al. 2020	Physical Activity Programs during Pregnancy Are Effective for the Control of Gestational Diabetes Mellitus
	Bielefeld et al. 2020	The Effects of Legume Consumption on Markers of Glycemic Control in Individuals with and without Diabetes Mellitus: A Systematic Literature Review of Randomized Controlled Trial
	Bao et al. 2020	The influence of home-based exercise on gestational diabetes: a meta-analysis of randomized controlled trials

Figure S1. Quality of included reviews assessed by Amstar 2.

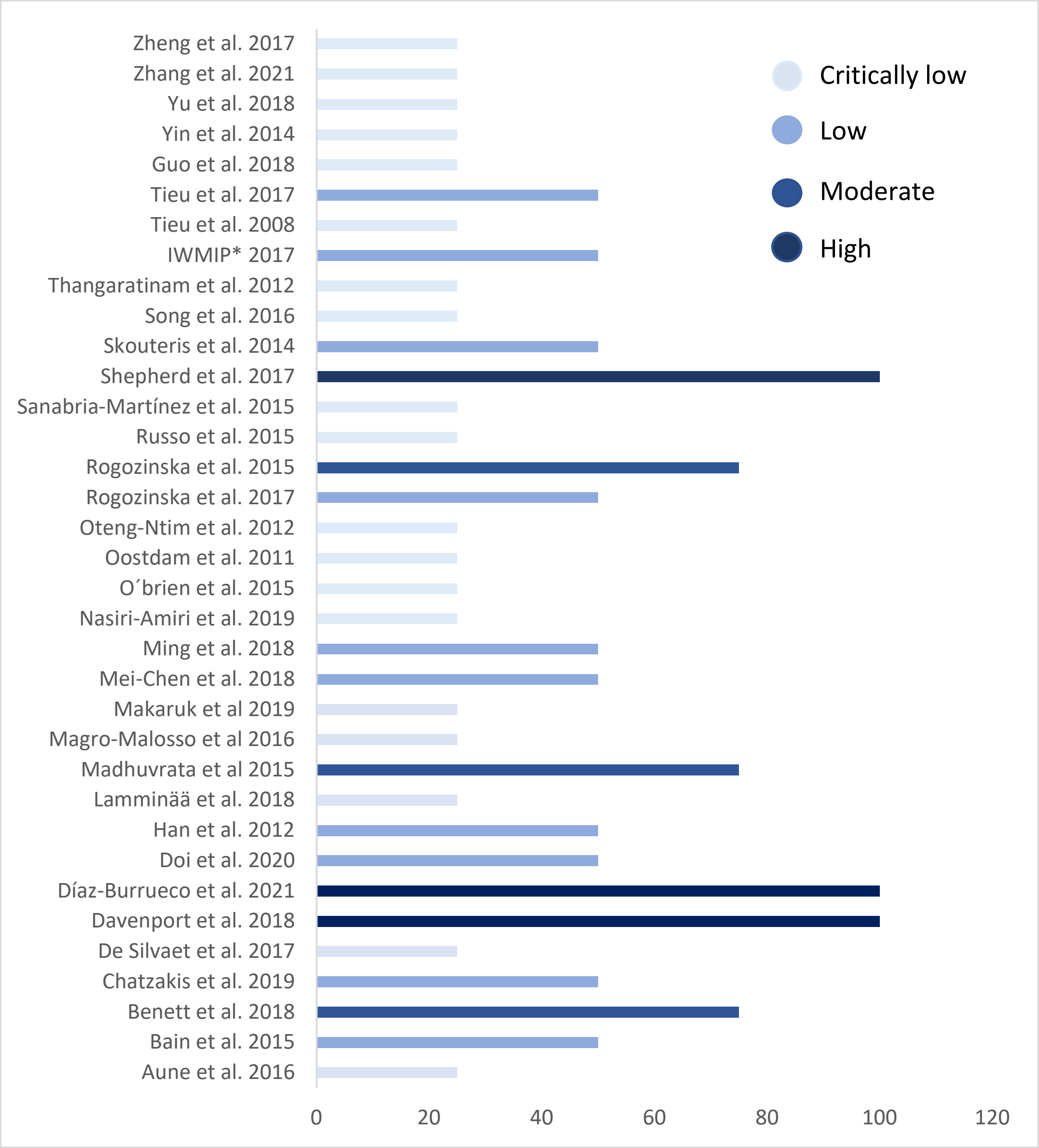

**The International Weight Management in Pregnancy*

Table S5. Overlapping assessment: Diet as only intervention (systematic reviews published since 2015)

**Systematic reviews with diet intervention published ≥ 2015 (pregnant women in general)**				
**Systematic review**	Rogozinska et al. 2015	Tieu et al. 2017	Bennett et al. 2018	Guo et al. 2018
**Primary studies**				
Bruno et al. 2016	0	0	0	1
Clapp et al. 2002	0	1	0	0
Fraser et al. 1983	0	1	0	0
Korpi-Hyovalti et al. 2012	1	0	1	1
Laitinen et al. 2009	0	1	0	0
Luoto et al. 2010	1	0	0	1
Liao et al. 2012	0	0	1	0
Markovic et al. 2016	0	1	0	1
McCarthy et al. 2016	0	0	1	1
Moses et al. 2006	0	1	0	0
Moses et al. 2014	0	1	0	1
Quinlivan et al. 2011	1	1	0	1
Thornton et al. 2009	1	1	1	1
Vitolo et al. 2011	0	1	0	0
Vesco et al. 2014	0	0	0	1
Walsh et al. 2012	1	1	1	1
Wolff et al. 2008	1	1	1	0
Xiao et al. 2015	0	0	1	0
Ye et al. 2016	0	0	1	0
Zhang et al. 2013	0	0	1	0
Zhang et al. 2015	0	0	0	1
**Total** (Nº of studies per reviews)	6	11	9	11
**Grand Total (N)**	37			
**Rows (r)**	21			
**Columns (c)**	4			
**Corrected covered area (CCA)**	**25.39%**			

Table S6. Overlapping assessment: Physical activity as only intervention (systematic reviews published since 2015)

**Reviews with physical activity intervention published ≥ 2015 (pregnant women in general)**											
**Systematic reviews**	Russo et al. 2015	Sanabria-Martínez et al. 2015	Aune et al. 2016	Zheng et al. 2017	Da silva et al. 2017	Bennett et al. 2018	Davenport et al. 2018	Guo et al. 2019	Yu et al. 2018	Makaruk et al. 2019	Díaz-Burrueco et al. 2021
**Primary studies**											
Barakat et al. 2009	0	1	0	0	0	1	1	0	0	0	0
Barakat et al. 2011	0	0	0	0	0	1	0	0	0	0	0
Barakat et al. 2012	1	1	1	0	1	0	1	0	0	1	0
Barakat et al. 2013	1	1	1	1	1	1	1	1	1	1	0
Barakat et al. 2014	1	1	1	0	1	0	1	1	0	1	0
Barakat et al. 2014	0	1	0	0	1	1	0	0	0	0	0
Barakat et al. 2016	0	0	0	0	0	1	1	1	0	1	1
Barakat et al. 2019	0	0	0	0	0	0	0	0	0	0	1
Bisson et al. 2015	0	0	0	0	0	1	1	1	0	0	1
Callaway et al. 2010	1	0	1	0	0	0	1	1	0	0	0
Cordero et al. 2015	0	0	1	0	1	0	1	0	1	1	1
da Silva et al. 2017	0	0	0	0	0	0	0	0	0	1	1
Daly et al, 2017	0	0	0	0	0	0	0	0	0	0	1
Elden et al. 2008	0	0	0	0	0	0	1	0	0	0	0
Garnaes et al. 2016	0	0	0	0	0	0	1	1	0	1	1
Guelfi et al. 2016	0	0	0	1	0	0	1	1	1	0	1
Ko CW et al. 2014	1	0	1	0	0	0	1	1	0	0	0
Kong et al. 2014	0	0	0	0	0	1	1	1	0	0	0

Nobles et al. 2015	0	0	1	1	0	0	1	1	1	0	0
Okido et al. 2015	0	0	0	0	0	0	1	0	0	0	0
Oostdam et al. 2012	1	0	1	0	0	1	1	1	0	1	0
Perales et al. 2020	0	0	0	0	0	0	0	0	0	0	1
Price et al. 2012	1	1	1	0	1	0	1	1	0	0	0
Rakhshani et al. 2012	0	0	0	0	0	0	0	1	0	0	0
Renault et al. 2014	1	0	1	0	0	1	1	1	0	0	0
Rodriguez et al. 2012	0	0	0	0	1	0	1	0	0	0	0
Ruiz et al. 2013	0	1	0	0	1	1	1	1	0	0	0
Seneviratne et al. 2016	0	0	0	0	0	0	1	1	0	0	1
Simmons et al. 2015	0	0	0	0	0	0	1	0	0	0	0
Simmons et al. 2016	0	0	0	0	0	0	1	0	0	0	0
Stafne et al. 2012	1	0	1	1	1	0	0	1	1	1	0
Tomić et al. 2013	1	1	1	0	1	0	1	1	0	0	0
Ussher et al. 2015	0	0	0	0	0	0	1	0	0	0	0
Wang et al. 2016	0	0	0	1	0	0	1	1	0	0	0
Wang et al. 2017	0	0	0	0	0	0	0	0	1	1	1
**Total** (Nº of publications per review)	10	8	12	5	10	10	26	19	6	10	11
**Grand Total (N)**	127										
**Rows (r)**	35										
**Columns (c)**	11										
**Corrected covered area (CCA)**	**26.28%**										

Table S7. Overlapping assessment: Physical activity as only intervention (systematic reviews published since 2015 in high-risk women)

Systematic reviews with physical activity intervention published ≥ 2015 (pregnant women at high risk)						
	Madhuvrata et al. 2015	Ming et al. 2018	Mei-Chen et al. 2018.	Chatzakis et al. 2019.	Nasiri-Amiri et al. 2019.	Doi et al. 2020.
**Primary studies**						
Barakat et al. 2012	0	1	0	0	0	0
Barakat et al. 2013	0	1	0	0	1	0
Barakat et al. 2014	0	1	0	0	0	0
Barakat et al. 2014	0	1	0	0	0	0
Bisson et al. 2015	0	0	1	1	0	1
Callway et al. 2010	1	0	1	1	1	1
Cordero et al. 2015	0	1	0	0	0	0
Daly et al. 2017	0	0	1	1	1	0
Dekker 2015	0	0	0	1	0	0
Garnæs et al. 2016	0	0	0	1	1	1
Garnaes et al. 2017	0	0	1	0	0	0
Guelfi et al. 2016	0	0	0	0	0	1
Hui et al. 2014	0	0	0	0	0	1
Kong et al. 2014	0	0	1	1	0	1
Nascimento et al. 2011	0	0	0	1	0	0
Nobles et al. 2015	0	0	0	0	0	1
Ong et al. 2009	1	0	0	1	0	0
Oostdam et al. 2012	1	0	1	1	1	1
Renault et al. 2014	0	0	1	1	0	1
Ruiz et al. 2013	0	1	0	0	0	0
Seneviratne et al. 2016	0	0	1	1	1	1
Simmons et al. 2016	0	0	0	0	1	0
Simmons et al. 2017	0	0	1	1	0	0
Stafne et al. 2012	0	1	0	0	0	0
Tomic et al. 2013	0	1	0	0	0	0
Wang et al. 2016	0	0	0	0	1	0
Wang et al. 2017	0	0	1	1	0	1
**Total** (Nº of publications per review)	3	8	10	13	8	11
**Grand Total (N)**	53					
**Rows (r)**	27					
**Columns (c)**	6					
**Corrected covered area (CCA)**	**19.25%**					

Table S8. Overlapping assessment: Physical activity as only intervention (Systematic reviews published before 2015)

**Systematic reviews with physical activity intervention published < 2015 (pregnant women in general)**			
**Systematic reviews**	Oostadam et al. 2011	Han et al. 2012	Yin et al. 2014
**Primary studies**			
Barakat et al. 2009	1	0	1
Barakat et al. 2011	0	1	1
Callway et al. 2010	0	1	1
Hopkins et al. 2010	1	1	0
Ong et al. 2009	1	1	0
Price et al. 2012	0	0	1
Rakhshani et al. 2012	0	0	1
Stafne et al. 2012	0	1	1
**Total (**Nº of publications per review)	3	5	6
**Grand Total (N)**	14		
**Rows (r)**	8		
**Columns (c)**	3		
**Corrected covered area (CCA)**	**37.50%**		

Table S9. Overlapping assessment: Mixed intervention (systematic reviews published since 2015)

**Systematic reviews with mixed intervention published ≥ 2015 (pregnant women in general)**					
	Rogozinska et al. 2015	Shepherd et al. 2017	Bennett et al. 2018	Davenport et al. 2018	Guo et al. 2018
**Primary Study**					
Bogaerts et al. 2013	1	0	1	0	1
Bruno et al. 2016	0	1	0	0	0
Dodd et al. 2014	1	1	1	1	1
Guelinckx et al. 2010	1	0	0	1	0
Harrison et al. 2011	1	0	0	0	0
Harrison et al. 2013	0	1	1	1	1
Hawkins et al. 2015	0	0	1	0	0
Herring et al. 2016	0	1	0	0	1
Hui et al. 2006	0	0	0	1	0
Hui et al. 2011	1	1	1	1	1
Hui et al. 2014	0	1	1	1	1
Jeffries et al. 2009	1	0	0	0	0
Jing et al. 2015	0	1	1	1	1
Jiang et al. 2015	0	0	1	0	0
Koivusalo et al. 2016	0	1	0	1	1
Korpi-Hyovalti et al. 2011	0	1	0	0	0
Luoto et al. 2011	0	1	1	1	1
Petrella et al. 2013	0	1	0	0	0
Petrella et al. 2013	1	0	1	1	1
Phelan et al. 2011	1	1	1	1	1
Polley et al. 2002	1	1	1	1	1
Poston et al. 2013	1	1	0	0	0
Poston et al. 2015	0	1	1	1	1
Rauh et al. 2013	0	1	0	1	1
Renault et al. 2014	1	0	1	1	1

Sagedal. et al. 2017	0	1	1	1	1
Simmons et al. 2015	0	0	0	1	0
Simmons et al. 2016	0	0	0	1	0
Sun et al. 2016	0	0	0	0	1
Smith et al. 2016	0	0	0	1	0
Stafne et al. 2012	0	0	0	1	0
Vesco et al. 2013	1	0	0	0	0
Vesco et al. 2014	0	0	1	1	0
Vinter et al. 2011	1	1	1	1	1
Wang et al. 2015	0	1	0	0	0
Wang et al. 2016	0	0	1	0	0
Zhang et al. 2015	0	0	1	0	0
**Total** (Nº of publications per review)	13	19	19	22	18
**Grand Total (N)**	91				
**Rows (r)**	37				
**Columns (c)**	5				
**Corrected covered area (CCA)**	**36.48%**				

Table S10. Description of physical activity intervention in included systematic reviews

Exercise						
**Authors**	**Publication year**	**Start and end of intervention**	**Type of intervention**	**Intensity**	**Frequency**	**Duration**
Díaz-Burrueco et al.	2021	Interventions started between 8-18 weeks gestation, and 2 studies started at 36-38 GW. The end was generally between 35-39 gestational weeks, x1 to delivery, x1 6 weeks post-partum	x6 supervised exercise including (aerobic, strengthening, stretching guided exercises), x3 Cycling program, x1 water exercise, x1 walking		The frequency was 3 times/ week in 10 studies, in one was 3-5 times/week	in 6 studies the duration was 60 min, x1 50-55min, x1 55min, x1 20-60min., x1 15-30min, x1 30 min
Doi et al.	2020	5/11 RCTs health care facility supervised exercise program. 6/11 supervised facility outside health care facility. In 7 studies, recruitment was prior to the 16 gestational weeks. In 4 studies have been up to 20 weeks. Intervention continued till approximately 36 weeks.				
Guo et al.	2019	Interventions started from 7 to 20 weeks of gestation				
Nasiri-Amiri fatemeh et al.	2019	In 2 studies, exercise began in the first trimester and continued until delivery. In three studies, exercise activities began in the second trimester and lasted until 34–37 weeks of gestation. In two studies, exercise	Studies included aerobic exercise (x3), aerobic and resistance (x1), aerobic and strength (x1), only resistance (x1), pelvic floor exercise (x1).	The intensity level of exercise activities was low to moderate in two studies, moderate to high in three studies, and moderate in a study.	In 1 study exercise was repeated 2x/week. In 4 was repeated 3x/week. In 1 study was daily repeated during a week. In	In all studies the duration was between 15-60min.

		activities started less than 17 weeks and continued up to 6 weeks after delivery.			1 study repeated 3-5x/ week.	
Makaruk et al.	2019	Interventions started between 6-22 gestational week, and continued till 34-delivery	The most frequent exercise was aerobic, resistance and strength exercise. And less frequently pelvic floor exercise and balancing exercise. For warm-up and the cool-down walking, stretching was the most frequent. Cycling and swimming was performed less frequently	Mostly the intervention was moderate	The frequency was 3 times/week. Except in one trial it was 2 times/ week.	In 5 studies the duration of the mean part of exercise sessions was between 20-35, and in 5 studies was between 40-50 min. And in 1 study, Between 30-60min (individually prescribed)
Du et al.	2019	In five studies, the mean gestational age at recruitment was 11.4 ± 2.7 week. Three recruited women before 20 weeks.<br>In three, the recruitment of women was realized before 20 weeks, 1 at 18 weeks, one before 16 weeks28; two at 12 weeks, and one at 11-14 weeks of gestation. In seven studies the intervention was continued until delivery, four studies to the 32-36 week's gestation, and two ended the intervention at the 28 week's gestation.	In three studies, the exercise program was "stationary cycling". In another three studies the program was "aerobic strength and muscle exercises". In two studies the activity was "walking for 11 000 steps or at least 30 minutes daily". Three studies had a "mixed method of stationary cycling, treadmill walk, and muscle exercise". And two studies used a "personalized exercise plan".		The frequency of the "stationary cycling" program was 3/week. The frequency of "aerobic strength and muscle exercises" was 2-3 times/week. For the mixed method was 1-3 times/week	The duration of the "stationary cycling" program was 30min/session. Duration of "aerobic strength and muscle exercises was 60 min/ session. And for the mixed method was about 50min/ session
You et al.	2018		Three studies reported a supervised cycling program. The intervention was based on the		The cycling program was realized 3 times/ week.	

			American college of obstetricians and gynecologists guidelines in another three studies.			
Bennett et al.	2018	Recruitment was realized mostly < 12-20 gestational week.	Intervention consisted of group exercise classes, including aerobic, muscle strength, flexibility, toning, and resistance exercises. An aquatic session and light resistance activity was mentioned (x1) and an unsupervised walking program (x1).	The intensity was light-moderate (x1), moderate exercise (x2), light resistance activity (x1)	The frequency is mainly 3 times/week.	The duration of exercise ranged from 35 to 60 min per session
Davenport et al.	2018		Seven RCTs with exercise the only intervention including aerobic exercise alone, two resistance training alone, one pelvic floor muscle training alone, and 11 RCTs including various types of exercise.		Frequency of exercise ranged from 1 to 7 days/week.	The duration of exercise ranged from 10 to 90 min per session
Ming et al.	2018	Seven trials started in the first trimester and continued to the end of the third trimester. Only one trial spanned the 20th through 36th weeks of gestation	Only one study includes aerobic exercise alone as the intervention, two studies included two types of interventions ("aerobic and resistance exercise"; and "two on land and one as aquatic exercise"). The rest of the studies included 3 types of exercise of the following (aerobics, stretching, resistance, mobilization, toning, flexibility, and muscle strength.)	light to moderate intensity	3 times/ week except in one study was 1 / week	the period ranged from 35-60min
Zheng et al.	2017		Physical activity based on American College of Obstetricians and Gynecologists (ACOG) guidelines. In two	The intensity of exercise in the included studies were different	The frequency of the supervised cycling program was 3 times/ week	The duration time of exercise in the

			studies the intervention was a supervised cycling program			included studies were different
Da Silva et al.	2017	Nine studies started between the 6-10 gestational week. And six studies started as minimum in the 12 gestational weeks. Two studies were continued to delivery, eight in the third trimester, one between the 20-36 week's gestation, and three did not mention the end's intervention date.	All interventions comprised a structured exercise program. The majority of trials included aerobic exercises and muscle resistance or strength training. Other types of activities like walking or stretching were reported.	Most included moderate-intensity physical activities: five trials reported a light to moderate intensity, seven moderate, and one moderate to vigorous. Intensity was not reported in two studies.	3 times/ week reported in 11 of studies, in 4 studies the frequency was. 1h, 2h and 4h per week.	The duration of sessions varied between 20 and 70 min. Only one study reported less than 30 min.
Madhuvrata et al.	2015	Started between 12-36 week's gestation	Individualized exercise programs with an energy expenditure. Session of aerobic and strength exercises under supervision of trained physiotherapist. 10 weeks of home-based supervised cycling exercise	Started with a 50–60% Hr_{max} and progress to 60–70%,	2-3 times/week	25-60min (data provided for 2 trials)
Sanabria-Martínez et al.	2015	Programs were conducted throughout pregnancy in 7 studies, and from the second trimester to the end of pregnancy in 6 studies	Supervised interventions consist in aerobic exercises (x11) resistance, toning, flexibility, and strength exercises (x7) weight training (x2). Strength exercises with muscles of the pelvic floor (x4).	In the majority of trials (x8) the intensity was moderate, and in four studies was light to moderate.	The frequency in the majority of trials (nine studies) was three times/week. In two trials was five times/ week. The minimum was two sessions in one trial.	Sessions lasted between 15 and 60 minutes.
Russo et al.	2015	The majority of studies enrolling participants at less than 16 weeks of gestation. With a variability between 6-8 and 18-22 weeks.	The intervention consisted of a group exercise session, except in four trials. All of the interventions included an aerobic component (walking, land or		Mostly the frequency was about 3 times/ week	Duration of exercise ranged from 35-60 min

			water aerobics or both, cycling), and four included an anaerobic component (strength training and balance exercises)			
Yin et al.	2014	Beginning gestation weeks (6–18 weeks)	The intervention consists of an individualized plan, regular exercise advice and paper-based instructions. In only one study, trials included aerobic exercise, resistance exercise, aerobic sessions, aquatic activities, stretching, and balance exercises. One study also included a yoga session.	Light intensity (x1)	3-4 times/ week	35-45
Han et al.	2012	6-9 GW to 38-39 GW, 12-week gestation.	The intervention consisted of home-based supervised exercise (x2), supervised sessions, individualized exercise plan (by face-to-face visits and phone calls), and paper-based diaries, 2 home exercises, and one group session. Fitness specialist and obstetrician, physiotherapist conducted interventions. Exercises consist of: aerobic (x4) sessions, cycling (x2), strength training, balance exercise, and aquatic activities. (one study provided diet recommendation and pelvic floor muscle exercise to both groups intervention and control)	Light-moderate (x2)	3-5 times/ week	35-60 min

Table S11. Description of diet intervention in included systematic reviews

Diet							
**Authors**	**Publication year**	**Start and end of intervention**	**Type of intervention**	**Visits**	**Service provider**	**Guide and recommendations**	**Personalized recommendations**
Zhang et al.	2020		In two studies, this consisted of high consumption of fruits and vegetables, nuts, less intake of meat and moderate to high on fish. All studies include virgin olive oil, and two include nuts,				
Guo et al.	2019	Interventions started from 7 to 20 weeks of gestation					Individual dietary advice (x1), individual dietary assessment (x1)
Bennett et al.	2018	recruitment was realized mostly < 12-20 gestational week.	Intervention consisted of dietary advice for healthy eating. The recommendation mostly favored a balanced diet with a percentage recommendation for each nutrient, low-GI and energy-restricted diet was encouraged. And trials encouraging self-weighing.	Counseling usually performed in an individual session (3-10 visits). One trial completed the face-to-face visit by 1 phone call, and one trial relied on group education sessions. In other trials, monitoring was realized every 4 weeks, and more frequently for some participants.	dietitian (x2)	Australian Guide to Healthy Eating was used in two trials, Danish dietary recommendations, IOM recommendations, Chinese Society of Nutrition Pregnancy Guidelines.	A personalized intervention is provided in most studies: individualized intake restrictions, women provided with food products, diet-based on the participant's weight, counseling according to their baseline nutritional status.
Lamminpää et al.	2018	-	-	-	-	-	-
Tieu et al.	2017		Four studies compared low-glycemic index (GI)	The frequency was very different between studies.	Interventions were	Different guidelines were used:	Energy intake was restricted based on

			with moderate- to high-GI dietary advice; and one compared specific (high-fiber focused) with standard dietary advice.	Some made 10 one-hour consultations. Others, 3 times during pregnancy. And two hours sessions	conducted mainly by dietitian, and also by research dietitian and food technologist.	Danish dietary recommendation, Nordic nutrition recommendation, general healthy eating guidelines, Australian guidelines from the National Health and Medical Research Council, Conventional nutrition guidelines,	individually estimation

Table S12. Description of mixed intervention in included systematic review

Combined intervention									
**Authors**	**publication year**	**started and end of intervention**	**type of intervention**	**intensity**	**Frequency/ visit**	**duration**	**services provider**	**guide and recommendation**	**personalized recommendation**
Guo et al.	2019	Interventions started from 7 to 20 weeks of gestation					trained graduate, dietitian		
Bennett et al.	2018	recruitment was realized mostly < 12-20 gestational week.	Advice regarding physical activity and diet was realized through regular clinic visits often supported by phone calls. **Exercise:** Interventions consisted of sessions at the gym (x3), physical activity DVD, recommendation to increase physical activity to 11000 steps/day, walking. **Diet:** Nutritional education sessions, brochure regarding diet, group session recording energy intake and intake recommendation, meal	Moderate (x2)	3-5 sessions plus 4-6 phone calls. **Exercise:** Recommendation of physical activity frequency is around 3 times/week **Diet:** 3-6 face-to-face consultation plus phone calls. Counseling varied between every two weeks alternating outpatient and phone calls and 4 dietary counseling sessions. Two trials reported the duration (one hour counseling and 1.5 nutrition education lecture).	30-60 min	Mostly the intervention was realized by dietitians (5 studies), in 4 studies was realized by dietitians and gynecologists or trained trials nurses or graduate students. In other studies was realized by exercise physiologist, 1 trained graduate student, nurse and physiotherapist, masters and doctoral with nutritional training, health trainer, trained researchers.	DASH diet.	

			plans, recipes, and snack ideas were provided, individual change behavior session, balanced diet, energy-restricted diet, encouraging low-GI diet, and reduced saturated fat. Dietary advice included portion sizes, regular meals, snacking, increasing intake of water, fruit and vegetables. cooking class.						
Shepherd et al.	2017	One study mentioned that the interventions started at 22 week's gestation to 36 week's gestation. In other studies, the intervention was initiated in the first trimester. In one study, the intervention began at 19 weeks' gestation	**Exercise:** The intervention in the majority of studies consisted of an intensive lifestyle intervention, or intensive exercise program. One study consisted of a recommendation that women increase activity to walking 5000 steps daily. Four studies recommended min 30 min daily walking or the	Seven studies included moderate-intensity exercise, one mild exercise, and one mild to moderate	**Exercise:** The frequency varied between 3 -7 times per week. 3 times a week was the most frequent in the recommendation.<br>**Diet:** The majority of intervention was realized in one-on-one visit (9 studies). 6 studies one-on-one visit with a weekly or monthly group	Session's duration recommended is generally 30 min with some variation that can reach 40-45 min in		Frequently the interventions are based on some guide: Institute of Medicine (IOM) guidelines (4 studies) and American College of Obstetricians and Gynecologists guidelines for	Few studies reported a personalized intervention: personalized monitoring (2 studies), individually-tailored diet advice (2 studies)

			most frequent possible. And in one study, women received a free six-month gym membership. **Diet:** The most frequent intervention was a hypocaloric or low-glycemic diet. The intervention was conducted in different ways: phone calls that provided diet advice or structured meal plans, written education materials/reminders, notebook for monitoring and exercise recommendation.		session, phone session, or distribution of digital scales.	some intervention		gestational weight gain,	
Song et al.	2016	20 Trials initiated the intervention at or before 15 gestational week, and 8 after the 15th gestational week.					dietitian,		
Rogozińska et al.	2015	All the interventions were commenced before 28 weeks at	**Exercise:** Recommendation to increase the amount of walking and	Intensity was reported	**Exercise:** 3-5 times/ week. **Diet:** Different type of visit was	The duration was reported	A dietitian and nutritionist mostly provided the sessions. Many times	The most mixed approach intervention was	

		varied time points in the first or second trimester.	incidental activity, two trials just recommended walking, and trials recommend different types of activities: swimming, aerobic, stretching, strength exercise. One trial conducted an indoor training with light weights, elastics band, balance, and aerobics exercises. **Diet:** Change of habitual diet in interventions groups more frequently by recommendations for a healthy and balanced diet. Increase fiber, increase low-GI food. A study used a simple behavioral change using a social cognitive theory.	as moderate in just one trial.	reported in 3 trials: one-to-one contact session, four sessions of behavioral change and lifestyle interventions. A session every 2 weeks.	for two studies, 30-45 min, and 30-60 min, respectively.	accompanied by physiotherapists, or nurses. Masters and doctoral level staff with training in nutrition or clinical psychology.	based on the official National Dietary Recommendations. The Institute of Medicine (IOM) guidelines and Danish recommendation were also used.	
Madhuvrata et al.	2015	15-35 week's gestation	**Exercise:** In general, intervention was based on encouraging physical activity by	Moderate exercise recommended	**Diet:** 3 group sessions (5 women/1h), one face-to-face	30 min most days	Nurse (x3) or doctor, Licensed fitness trainers and registered dietician, Masters or Doctoral students, food	Official National Dietary recommendation, Danish	Individual estimation of Energy requirements,

			aerobic and aquafit classes provided by a specialist and written and oral instruction, or encouraging walking. A study provided free membership to a fitness center for 6 months with closed training classes 1h every week and use of a pedometer. **Diet:** Phone calls, brochure which provided advice on nutrition, balanced healthy diet, recommendation for energy intake, automated postcards that promote healthy eating.	in two trials			technologist and clinician physiologist, study coordinator, exercise physiologist, trained dietitian, and primary care provider.	recommendations.	individually-tailored diet advice.
Skoutris	2014	8-12 at 20-28 gestational week	Individual session in most trials, the intervention consisted of verbal counseling with written support education in some trials. **Exercise:** Seven studies encouraged physical				Mostly dietitian (x5), clinic nurse, nutritionist	Health Canada guidelines, Prenatal nutrition guidelines	

			activity, one mentioned pelvic floor exercises as a specific exercise. **Diet:** Food Choice map, Information, and recommendation on healthy eating.						
Oostdam et al.	2011	Varying start dates	**Exercise:** Supervised exercise, group session, or brochure recommendation consist in encouraging physical activity especially toning exercise, cycling (data presented separately) brochure and active counseling session, or phone calls consist in the promotion of balanced diet recommendation, high dietary fiber, and low intake of sucrose, restricted energy diet LGI diet, one trials consist in self-monitoring	Moderate exercise, light resistance	3-5 times/ week				Personalized plan and recommendation (x1)
